# Calcium Reduces Liver Injury in Mice on a High-Fat Diet: Alterations in Microbial and Bile Acid Profiles

**DOI:** 10.1371/journal.pone.0166178

**Published:** 2016-11-16

**Authors:** Muhammad Nadeem Aslam, Christine M. Bassis, Li Zhang, Sameer Zaidi, James Varani, Ingrid L. Bergin

**Affiliations:** 1 The Department of Pathology, The University of Michigan, Ann Arbor, MI, 48109, United States of America; 2 The Department of Internal Medicine, The University of Michigan, Ann Arbor, MI, 48109, United States of America; 3 Michigan Regional Comprehensive Metabolomics Resource Center, The University of Michigan, Ann Arbor, MI, 48109, United States of America; 4 The Unit for Laboratory Animal Medicine, The University of Michigan, Ann Arbor, MI, 48109, United States of America; Ospedale Casa Sollievo della Sofferenza, ITALY

## Abstract

A high-fat “Western-style” diet (HFWD) promotes obesity-related conditions including non-alcoholic steatohepatitis (NASH), the histologic manifestation of non-alcoholic fatty liver disease (NAFLD). In addition to high saturated fat and processed carbohydrates, the typical HFWD is deficient in calcium. Calcium-deficiency is an independent risk factor for many conditions associated with the Western-style diet. However, calcium has not been widely evaluated in the context of NAFLD. The goal of the present study was to determine if dietary calcium supplementation could protect mice fed a HFWD from NAFLD, specifically by decreasing non-alcoholic steatohepatitis (NASH) and its down-stream consequences. Male C57BL/6NCrl mice were maintained for 18-months on a HFWD containing dietary calcium at either 0.41 gm/kg feed (unsupplemented) or 5.25 gm/kg feed (supplemented). Although there was no difference in body weight or steatosis, calcium-supplemented mice were protected against downstream consequences of hepatic steatosis, manifested by lower inflammation, less fibrosis, and by lower overall histologic NAFLD activity scores (NAS). Calcium supplementation correlated with distinctly segregating gut fecal and cecal microbial communities as defined by 16S rRNA gene sequence. Further, calcium supplementation also correlated with decreased hepatic concentration of the major conjugated murine primary bile acid, tauro-β-muricholic acid (as well as a decrease in the parent unconjugated bile acid). Thus, calcium was protective against progression of diet-induced hepatic steatosis to NASH and end-stage liver disease, suggesting that calcium supplementation may effectively protect against adverse hepatic consequences of HFWD in cases where overall diet modification cannot be sustained. This protective effect occurred in concert with calcium-mediated gut microbial community shifts and alterations of the hepatic bile acid pool.

## Introduction

Non-alcoholic fatty liver disease (NAFLD) is a common consequence of obesity and metabolic syndrome [1,2] and is rapidly increasing in Western society [3–5]. At its earliest stages, steatosis (fatty infiltration of the liver), is the major manifestation. While steatosis is generally well-tolerated, in some individuals there is progression to non-alcoholic steatohepatitis (NASH), consisting of inflammatory foci and characteristic ballooning degeneration of hepatocytes. Ensuing cycles of hepatocellular injury and regeneration lead to fibrosis and, eventually, cirrhosis, which is an end-stage state characterized by widespread inflammation, fibrotic scarring and formation of regenerative hyperplastic nodules [5–8]. Additionally, in a subset of individuals, chronic NASH leads to hepatocellular carcinoma (HCC) [9–12]. NAFLD is rapidly becoming the most common cause of chronic liver injury in industrialized countries, in parallel with the increased consumption of a high fat, “Western-style” diet (HFWD) [3,4]. Dietary caloric restriction to reduce steatosis is a mainstay of NAFLD prevention, but its efficacy is hampered by poor compliance and potentially by early life metabolic and gut microbial alterations that have prolonged inhibitory effects on weight loss [4]. Since steatosis alone is well-tolerated, there may be significant benefit to pharmaceutical or nutraceutical interventions acting at downstream points in the progression from steatosis to the pathologic states of NASH and cirrhosis.

The progression of histological changes seen in NAFLD, including steatohepatitis with formation of regenerative hyperplastic nodules (RH) and/or hepatocellular neoplasms, has been demonstrated in mice fed a high content of saturated fat and excess calories [5,13–15]. In our own previous study, male C57BL/6NCrl male mice (but not female mice) maintained on a HFWD for up to 18 months developed liver tumors at a significantly higher rate and more rapidly than littermates fed a standard rodent chow diet (AIN76A) [13]. The HFWD is designed to mimic dietary habits prevalent in Western countries [16–18], and contains increased saturated fat and total calories as compared to a typical rodent chow diet. While the fat content is less than that of some other typical obesity-producing diets [15], the HFWD also has features of the choline deficiency amino acid-defined (CDAA) diet; namely a deficiency of methyl group donors (i.e., certain B vitamins, methionine, and choline) [19].

The low level of dietary calcium in Western-style diets may also play a role in the progression from steatosis to NASH. Studies from Europe, Australia and North America have shown that the Western-style diet does not provide an adequate amount of calcium for most individuals [20–24]. The most recent assessment by the Institute of Medicine Food and Nutrition Board [25,26], and implemented into the recent 2015 USDA dietary guidelines [27] calculated recommended daily allowances (RDA) for calcium of 1000–1200 for adult men and women, respectively. The Board concluded that a majority of individuals in virtually every age group did not meet the minimal RDA for calcium. Dietary calcium supplementation (either alone or in conjunction with specific nutrients) has been shown in some rodent studies to reduce weight and reduce metabolic features associated with a high fat diet, although not consistently [28–31]. Additionally, vitamin D deficiency (and thus disrupted calcium homeostasis) has been epidemiologically linked with the more severe manifestations of NAFLD [32].

The present study was undertaken to evaluate whether dietary supplementation with calcium alone could protect mice against HFWD-associated liver injury. In our previous study, male C57BL6/NCrl mice fed a HFWD but supplemented with a calcium-rich, multi-mineral-containing natural product had reduced non-neoplastic and neoplastic liver damage [13]. The current study was intended to separate the effects of calcium from multi-mineral supplementation. Male mice were used since NAFLD is more common in men [33] and this predominance is recapitulated in mouse models [13,34,35]. The C57Bl/6NCrl substrain was chosen since the B6N substrain has demonstrated higher body weight gains, higher percent body fat, and higher liver triglycerides in response to high fat intake than the B6J substrain [36]. This may be due to the known genetic divergence between B6N and B6J substrains, which can affect metabolic responses [37]. As part of the study, gut microbial populations and hepatic bile acids were evaluated for calcium-associated alterations, since dietary fat-related changes in gut microbes and metabolic profiles have been postulated as a contributing cause in obesity-associated diseases, including NASH [38–40]. In this study we demonstrated that calcium supplementation ameliorated liver injury and generated relevant alterations of gut microbes and bile acids but did not affect weight gain. Thus, the preventative effects of calcium supplementation appear to act downstream of steatosis and inhibit progression from benign hepatic fat accumulation to a pathologic state.

## Materials and Methods

### Animals

Male C57BL/6NCrl mice (Charles River Laboratories, Portage, MI) were used in this study. Mice were obtained at 3 weeks of age and housed for the duration of the study in standard polycarbonate cages within ventilated racks at a housing density of 5 per cage. Light:dark cycle was 12:12 hours and temperature and humidity were maintained at 72 ± 4°F and 30% to 70%, respectively. Animals were fed the study diets as described below. Food and water was available *ad libitum*. All of the procedures involving animals were reviewed and approved by the University (of Michigan) Committee on Use and Care of Animals in accordance with the NIH Public Health Service policy for the Humane Care and Use of Laboratory Animals.

### Diet groups

Two cohorts of male mice were included in this study. Mice (20 per group) were started at 4 weeks of age on a HFWD, prepared according to the formulation of Newmark et al [17] and used by us in prior studies [13,41–43]. The HFWD is a variant of a standard rodent chow diet, AIN76A, but contains 20% fat from corn oil as compared to 5% in AIN76A [13]. The overall calorie content in the HFWD is 4767 kcal per kg of diet, and the calcium level is 0.41 gm/kg diet compared to 3902 kcal per kg diet and 5.25 gm/kg diet in AIN76A. In this study, one group was maintained as a control (HFWD alone) while the other group received the same HFWD and additional calcium to bring the overall calcium level to 5.25 gm/kg diet as in standard AIN76A rodent chow. Thus the calcium level in the supplemented diet represented a “normal” (but not excessive) level for typical rodent diets, while the unsupplemented group was calcium-deficient. Diets were provided by Research Diets Incorporated (New Brunswick, NJ). The complete composition of the two diets is presented as Supplemental Materials (S1 Table).

### Experimental protocol and necropsy

Mice were maintained for 18 months on their respective diets. Animals were monitored at 2-day intervals throughout the maintenance phase and were weighed every two weeks. Animals were euthanized by CO_2_ inhalation at the end of the study and subjected to necropsy. Animals that died unexpectedly or displayed pre-defined criteria of moribund condition (per institutional guidelines) warranting euthanasia before the end of the 18-month period were also subjected to necropsy. Necropsy included gross examination of the intact livers. Masses were identified, photographed, and size was measured (diameter in two dimensions). Raw data for body and organ weights and clinical observations are in Supplemental Materials (S2 Table).

### Histological evaluation of non-proliferative lesions

Livers were removed at necropsy and fixed in 10% buffered formalin. Sections were processed to paraffin by routine histological methods and 4 μm sections were stained with hematoxylin and eosin. Sections were evaluated under light microscopy by a board-certified veterinary pathologist (ILB) for identification of proliferative lesions as well as severity scoring of lesions associated with steatohepatitis. Steatohepatitis parameters were scored and classified according to a standardized histological scoring system for NASH described by Kleiner et al [7] and previously applied to the histological scoring of mouse models of steatohepatitis [44,45]. Specifically, individual livers were evaluated for steatosis, lobular inflammation, and ballooning degeneration of hepatocytes, and given a score of (0–3) for steatosis and inflammation, and (0–2) for ballooning degeneration, using previously defined criteria [7,44,45]. An NAFLD activity score (NAS) was obtained by summing the individual parameter scores. Necrosis was scored separately as 0 (none), 1 (minimal: few or small foci), 2 (mild: multiple small foci), 3 (moderate: multiple larger foci), and 4 (coalescing or regionally extensive). In parallel, sections were stained with Sirius red for assessment of collagen deposition (i.e., fibrosis) and scored as 0–4 as previously described by Kleiner et al [7]. Raw data for histological scoring of non-proliferative lesions is available as Supplemental Materials (S2 Table).

### Histological evaluation of liver masses

Proliferative lesions were classified according to recently revised, standardized guidelines established by the International Harmonization of Nomenclature and Diagnostic Criteria for Lesions in Rats and Mice (INHAND) project [46]. This scoring system represents consensus criteria for histopathological lesions in rodents as established by the North American, European, British, and Japanese Societies of Toxicologic Pathology. Proliferative lesions were classified as regenerative hyperplasia (RH), hepatic adenoma (HA), or hepatocellular carcinoma (HCC). RH represents the proliferative response of hepatocytes to cell injury. HA and HCC represent benign and malignant hepatocellular neoplasms, respectively. Raw data for histological assessment of liver masses is available as Supplemental Materials (S2 Table).

### Serum chemistry and pro-inflammatory cytokines

Blood was obtained at euthanasia by cardiac puncture and separated by centrifugation within a standard serum separator tube. Serum chemistry markers associated with liver cytotoxicity (alanine aminotransaminase [ALT] and aspartate aminotransferase [AST]) or liver function (albumin, total protein, alkaline phosphatase [ALKP], and total bilirubin) were obtained. Serum calcium, creatinine, and glucose levels were also assessed. Serum chemistries were evaluated using a Liasys 330 Clinical Chemistry Analyzer (AMS Diagnostics, Weston, FL). All blood analysis procedures were performed according to standard operating procedures within the In Vivo Animal Core (IVAC) of the Unit for Laboratory Animal Medicine at the University of Michigan. Pro-inflammatory cytokines in serum were evaluated using a custom-designed commercially available magnetic bead-based kit (Bio-Plex Pro mouse cytokines, Bio-Rad Laboratories, Hercules, CA) per manufacturer’s instructions. Raw data for serum chemistry and serum cytokines is available as Supplemental Materials (S2 Table).

### Microbial sequencing

Microbial sequencing was performed in the University of Michigan Host Microbiome Initiative Microbial Sequencing Core Facility. DNA was isolated from fecal and cecal samples using a PowerMag™ Soil DNA Isolation Kit (MO BIO Laboratories, Inc., Carlsbad, CA) on an epMotion® 5075 TMX automated liquid handling system (Eppendorf, Hamburg, Germany) according to manufacturer instructions. The V4 region of the 16S rRNA gene was amplified and sequenced as described previously [47]. PCR reactions used 1 μl undiluted DNA, 1 μl of DNA diluted 1:10, 5 μl of DNA diluted 1:50, 1 μl of DNA diluted 1:50, or 1 μl of DNA diluted 1:100. Sequencing was performed on an Illumina® MiSeq sequencer (Illumina, Inc., San Diego, CA), as described previously[47]. 16S rRNA gene sequences were processed and analyzed using the mothur software package version 1.34.4, 1.35.1 and 1.36.1 according to the publicly available MiSeq SOP [48,49]. After sequence processing and alignment to the SILVA reference alignment [50] sequences were binned into operational taxonomic units (OTUs) based on 97% sequence similarity. For our analysis, 4693 sequences were subsampled from each sample. Yue Clayton theta (θ_YC_) distances (a metric that considers relative abundances of both shared and non-shared OTUs [51]) between communities were calculated and significant differences were determined by analysis of molecular variance (AMOVA) [52]. Principle coordinates analysis (PCoA) was used to visualize the θ_YC_ distances between samples. Taxonomic composition of the bacterial communities was determined by classifying sequences within mothur using a modified version of the Ribosomal Database Project (RDP) training set [53,54].

Shannon diversity indices were calculated for all samples within mothur and compared by two-tailed t tests. Microbial community profiles were compared within mothur by AMOVA, performed on θ_YC_ distance values. Differentially abundant OTUs between groups were identified by linear discriminant analysis effect size (LEfSe) [55] and were compared within mothur by non-parametric means (Wilcoxan Rank Sum). Comparisons of particular OTU abundances were performed by Mann Whitney U within R. Significance was defined as p < 0.05 after multiple comparisons correction. Sequence data obtained in this study are available within the NCBI Sequence Read Archive, Study accession number SRP083284.

### Bile acid analysis

Bile acids were analyzed in samples of the right median liver lobe, including gall bladder, by the Michigan Regional Comprehensive Metabolomics Resource (MRC^2^) facility, using modifications of previous methodology [56,57]. In brief, right median liver lobe and gall bladder were obtained at necropsy and snap frozen in liquid nitrogen, followed by storage at -80°C until analysis. Samples were prepared by a two-step procedure consisting of extraction and centrifugation in 100% ethanol followed by chloroform/methanol (1:1), with reconstitution of the dried extract in 50% methanol-in-water and stored at 4°C until analysis. Analysis was performed using an auto sampler at 4°C. Samples were separated using reverse phase liquid chromatography (RPLC) and bile acid quantitation was by mass spectrometry using electrospray ionization-triple quadrupole-multiple reaction monitoring (ESI-QQQ-MRM) methodology. Data were normalized to original sample weight/volume and analytes were reported as pg/mg. Absolute quantitation (μM concentrations of analytes) was obtained using appropriate internal standards. Targeted metabolomics (bile acid) data obtained in this study from liver and gall bladder are presented in Supplemental Materials as a S3 Table.

### Statistical evaluation

For histological assessment, individual parameter scores and the summary NAS scores for each group were compared by Mann-Whitney U. The incidence of proliferative liver lesions (neoplastic or regenerative hyperplasia) in the two groups was compared using the Chi-square test. Liver bile acids and serum chemistry were compared using unpaired 2-tailed student t tests with and without Holm-Sidak corrections for multiple comparisons, respectively. Serum cytokines were compared by Mann Whitney U. All statistical analyses were performed within the program GraphPad Prism, version 6.0 except microbial community analyses, which were performed using the programs mothur and R as described under microbial sequencing.

## Results

### Weight gain, liver weights, and survival

Male C57BL/6NCrl mice were started on a 20% fat HFWD (20 animals per group) with or without calcium supplementation at 4 weeks of age and continued for 18 months. Mice in both the supplemented (HFWD-Ca) and unsupplemented (HFWD) groups gained weight over the 18-month period. Mice in the HFWD-Ca group gained weight slightly faster than mice in the HFWD group, but by the end of the study, average weights were comparable (53 ± 2 versus 53 ± 6 grams for HFWD and HFWD-Ca, respectively) (S1 Fig). At endpoint, livers from the HFWD group mice were significantly heavier than livers from the HFWD-Ca group mice (3.2 ± 1.5 versus 2.3 ± 0.7 grams for HFWD and HFWD-Ca, respectively; p = 0.022 by student *t* test).

Over the course of the 18-month observation period, 3 of 20 animals in the HFWD group died prematurely with large liver masses. In contrast, 0 of 20 animals in the HFWD-Ca group died prematurely due to liver lesions. Three additional animals in the HFWD group and 5 animals from the HFWD-Ca group died or were euthanized prematurely due to unrelated causes (e.g. ulcerative dermatitis).

### NASH-related liver injury

**Fig 1:**
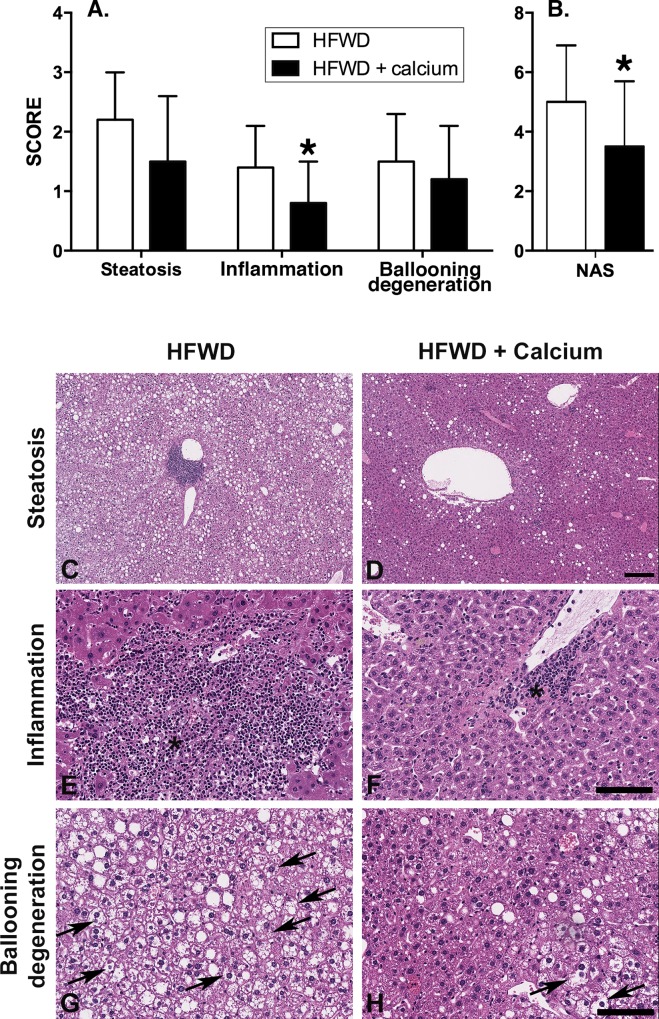
Histological assessment of NASH and liver injury. Histological sections were scored per established criteria (see Methods) in 19 of 20 HFWD mice and 20 of 20 HFWD-Ca mice. A. Individual components of NASH-related liver injury. B. NAS, calculated as the sum of the individual scores for the three component parameters. Inflammation and overall NAS were significantly lower in the HFWD-Ca group. (Mann-Whitney U, Steatosis, p = 0.1; Inflammation, p = 0.014; ballooning degeneration, p = 0.25; Overall NAS, p = 0.023). C-H: Representative histological features of NASH-related liver injury. Steatosis (C and D) consisted of macrovesicular cytoplasmic fat deposition distributed multifocally or diffusely. Multifocal lesions were most commonly in an azonal (random) or pericentral (zone 3) pattern. Inflammation (E and F) consisted of variably sized foci of infiltrating macrophages and neutrophils, most commonly in midzonal or pericentral (zone 3) areas (asterisk). Ballooning degeneration (G and H) consisted of markedly swollen hepatocytes with cleared cytoplasm and non-displaced nuclei (arrows). Hematoxylin and eosin staining. Scale bars = 200μm [C,D] and 100μm [E-H].

Histological sections of liver were scored separately for steatosis, inflammation, hepatocyte ballooning degeneration, and NAS (summary score) as previously described by Kleiner et al [7] (see Methods). All 20 mice in each group were evaluated, apart from one of the HFWD mice that died prematurely at 77 weeks. This mouse had no grossly observable liver masses but autolysis precluded histological scoring for non-proliferative features. Quantitative findings and representative histological sections are shown in Fig 1.

Mice in the HFWD-Ca group had decreased liver inflammation and decreased NAS (Fig 1; inserts A and B). Steatosis and ballooning degeneration were also lower for mice in the HFWD-Ca group than in the HFWD group (Fig 1A), although this did not reach statistical significance. Representative histological images are shown in Fig 1; inserts C-H. Steatosis, as anticipated for mice on a HFWD, was present to varying degrees in all mice, with severity ranging from mild to widespread (Fig 1; insert C and D). Inflammation, consisting of infiltrating neutrophils and mononuclear cells (macrophages and lymphocytes), was present multifocally, typically as small foci but occasionally larger confluent foci within areas of regenerative hyperplasia (Fig 1; insert E and F). Likewise, ballooning degeneration of hepatocytes, a marker of NASH-related cell injury, was frequently present, particularly in sections with the most prominent steatosis (Fig 1; insert G and H).

**Fig 2:**
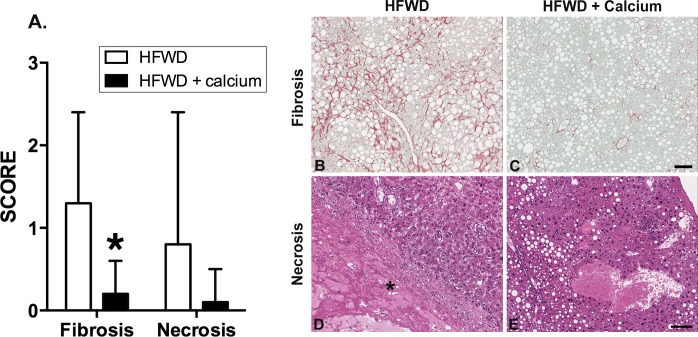
Histological scoring of fibrosis and necrosis. A. Fibrosis as indicated by Sirius red-staining for collagen was significantly lower in the HFWD-Ca group (asterisk, Mann-Whitney U, p = 0.0007). Necrosis assessed in H&E stained sections was also lower in the HFWD-Ca group, although this difference was not significant. B-E. Representative histological features. Fibrosis (B and C) varied from increased pericellular (“chicken wire” pattern) to pericentral (lobular) deposition in Sirius red stained sections. Necrosis (D) consisted of areas of devitalization (asterisk) that most commonly occurred within large regenerative hyperplastic nodules (RH) in HFWD mice. (E) depicts liver from a HFWD-Ca mouse with steatosis but no necrosis for comparison. Hematoxylin and eosin staining. Scale bar = 100μm.

Scores for fibrosis and necrosis were assessed separately from the NASH parameters and fibrosis was significantly lower for HFWD-Ca mice (Fig 2) Fibrosis appeared as increased pericellular collagen deposition (“chicken wire” pattern) (Fig 2; insert B and C) along with pericentral (lobular) collagen deposition in Sirius red stained sections. In HFWD-fed mice, large areas of necrosis (Fig 2; insert D) were present in 4 of 19 mice, in connection with large nodules of regenerative hepatocytes (n = 2) or hepatocellular carcinoma (n = 2). In three of these mice, necrosis was associated with liver lobe infarction and was considered the cause of death. In contrast, only 1 of 20 HFWD-Ca mice had hepatic necrosis and this was focal (one area only, detected only histologically). This animal survived to endpoint.

### Proliferative liver lesions in mice on the HFWD

Proliferative liver masses were identified grossly or histologically in 9 of 20 mice (45%) in the HFWD group, with a total of 11 masses in this group (2 mice had 2 lesions). In 3 of these mice, the masses were believed to be the cause of death, since the mice died prematurely and the masses were large with extensive necrosis affecting the entire lobe. In comparison, the HFWD-Ca group had liver masses in 6 out of 20 mice (30%), with 7 masses total (1 mouse had 2 lesions). None of the HFWD-Ca mice with liver masses died prematurely. The differences in total number of proliferative masses between groups were not significant.

**Fig 3:**
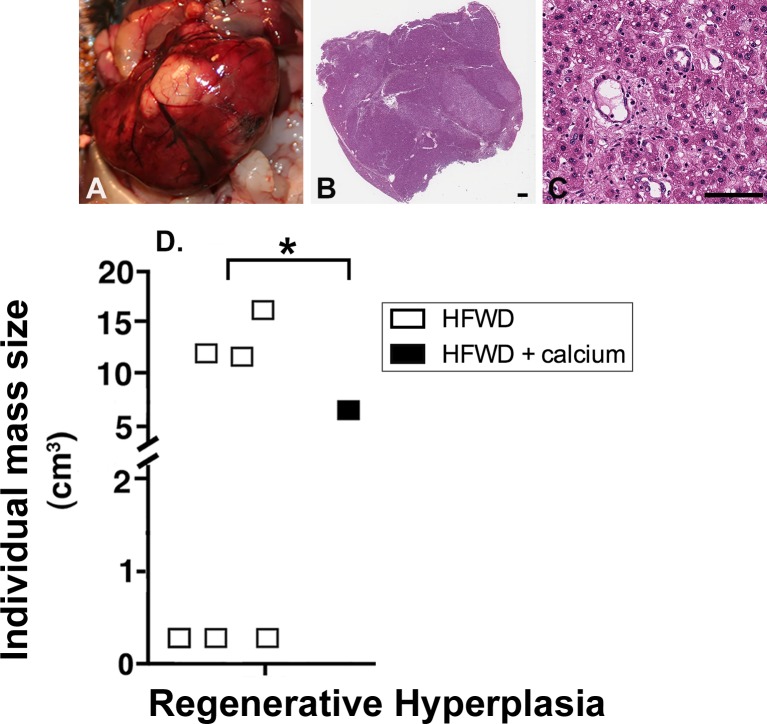
Regenerative hyperplasia (RH). Representative RH lesions are shown grossly (A) and histologically (B and C). RH nodules were distinguished from neoplastic masses by preservation of hepatic structures (ie. portal triads, central veins, 1–2 cell thickness of hepatic cords) within the area of proliferation. D. Frequency of RH nodules. Six of 20 HFWD mice had liver masses histologically classified as RH compared to 1 of 20 HFWD-Ca mice (Chi-square, 2-tailed, p = 0.0375). (Scale bars = 1mm [B] and 100μm [C]).

Histologically, masses consisted of non-neoplastic regenerative hyperplasia (RH), benign hepatic neoplasms (hepatic adenoma, HA), or malignant hepatic neoplasms (hepatocellular carcinoma, HCC). RH, although it often appeared to distort and enlarge the liver grossly (Fig 3A), was histologically distinguishable from neoplasia by preservation of hepatic structures (i.e., the presence of portal triads and central veins and hepatic cords having normal 1–2 cells thickness within the area of proliferation) (Fig 3B and 3C). There were significant differences between the two groups in the occurrence of RH. Of note, the HFWD mice had significantly higher occurrence of RH (6 of 20 mice) than the HFWD-Ca mice (1 of 20 mice) (p<0.0375 by Chi-square, two-tailed) (Fig 3D). Regenerative nodules are not a common aging lesion in mice and are a feature of cyclic hepatocellular injury and repair [46]. Regenerative hyperplasia can occur in the context of chronic NASH as part of the progression to cirrhosis. In contrast to RH, the number of mice with neoplastic lesions was comparable in both groups (n = 5/20 for HFWD, n = 6/20 for HFWD-Ca), reflecting a background (age-related) incidence of liver tumors.

### Serum biomarkers

Serum markers reflective of liver injury (AST and ALT), cholestasis (ALKP), or liver function (albumin, total protein and total bilirubin) were assessed in animals at euthanasia. Serum glucose, calcium, and creatinine (renal function) were also assessed. Serum total bilirubin (liver function marker) was significantly lower in the HFWD-Ca group than in the HFWD group (p = 0.0188, t test; Table 1). Elevated serum bilirubin can be indicative of impaired liver function. No significant differences were seen in other parameters. Results were similar when animals with liver masses were excluded (to separate the effects of diet from the potentially confounding effects of neoplasia or regenerative hyperplasia) (Table 1) or when all animals were included (ie. significantly lower serum bilirubin in the HFWD-Ca group, p = 0.0116, t test, S4 Table).

**Table 1:** Serum biomarkers in diet groups, excluding animals with liver masses^a^^,^^b^.

	HFWD (n = 11)^a^	HFWD-Ca (n = 10)^a^
AST (U/L)	149 ± 115	136 ± 45
ALT (U/L)	71 ± 22	73 ± 32
AlkP (U/L)	54 ± 23	36 ± 16
Total bilirubin (mg/dL)	0.14 ± 0.07	0.07 ± 0.05* (p = 0.0189)
Albumin (g/dL)	2.94 ± 0.60	2.97 ± 0.49
Total protein (g/dL)	5.66 ± 0.97	5.93 ± 0.76
Creatinine (mg/dL)	0.18 ±0.04	0.17 ± 0.03
Glucose (mg/dL)	252 ± 71	243 ± 38
Calcium (mg/dL)	9.30 ± 1.24	9.80 ± 0.63

^a^ 15 of 20 HFWD and 14 of 20 HFWD-Ca animals had no liver masses. Serum values were not available from 4 non-mass HFWD and 4 non-mass HFWD-Ca animals owing to post-mortem coagulation or insufficient sample.

^b^ Values are mean +/- S.D.

* significantly different by unpaired t tests, two-tailed without multiple comparisons correction

AST Aspartate Aminotransferase, ALT alanine aminotransaminase, ALKP alkaline phosphatase

Serum proinflammatory cytokines were also assessed in animals without liver masses (Table 2). MCP-1 was significantly lower in HFWD mice than in HFWD-Ca mice but other serum cytokines were not significantly different between the two groups.

**Table 2:** Serum cytokines in diet groups, excluding animals with liver masses^a^^,^^b^.

	HFWD (n = 8)	HFWD-Ca (n = 9)
IL-1β (pg/ml)	OOR<15	OOR<15
IL-6 (pg/ml)	5 ± 6	12 ± 26
KC (pg/ml)	55 ± 38	56 ± 24
MCP-1 (pg/ml)	166 ± 76	113 ± 82 (p = 0.03005)*
TNF-α (pg/ml)^c^	2.09 ± 2.49	0.94 ± 0.86

OOR< = Out of Range Below

^a^ 8 of the 11 non-mass HFWD and 9 of the 14 non-mass HFWD-Ca samples had sufficient serum left after clinical chemistry to conduct serum cytokine biomarker analysis.

^b^ Values are mean +/- S.D.

^c^ Values for TNF- α are based on 2 samples in each group, rest were OOR<0.33.

* significantly different by Mann Whitney U.

### Cecal and fecal microbial communities

**Fig 4:**
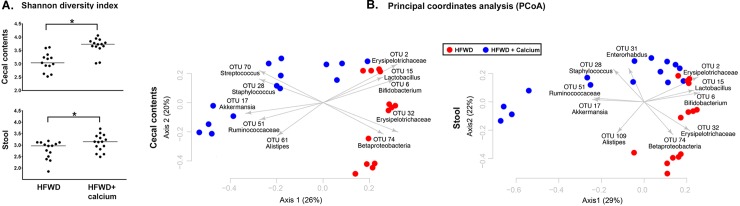
Increased gut microbial diversity in HFWD-Ca group and differential segregation of gut microbial communities. A. Increased microbial diversity in cecal (top) and fecal (bottom) microbial communities of HFWD-Ca group at study endpoint (18 months). B. PCoA depicting differential microbial segregation based on Illumina sequencing of the V4 region of 16S rRNA gene in cecal (left) and fecal (right) samples. Segregation between HFWD and HFWD-Ca groups was significant based on θ_YC_ distances (AMOVA, p<0.0001).

One possible mechanism for the protective effects of calcium against NASH-related pathology is a shift in gut microbial populations. To determine whether calcium supplementation altered the typical HFWD-associated gut microbiome, we performed Illumina sequencing of the V4 region of the 16S rRNA gene from cecal and fecal samples of 15 randomly selected mice in each group. Sufficient sequence amplification was obtained from all samples except one cecal sample in the HFWD group. After quality filtering, subsampling of a minimum of 4693 sequences resulted in identification of 5349 OTUs, defined on the basis of 3% sequence difference. There was greater gut microbial diversity in the HFWD-Ca group (Fig 4A). The HFWD-Ca supplemented group distinctly segregated from the unsupplemented HFWD groups with respect to both cecal and fecal microbial communities, as shown in Fig 4B, with the major OTUs driving this segregation. θ_YC_ distances between the HFWD and HFWD-Ca clusters were significantly different by AMOVA (p<0.001).

**Fig 5:**
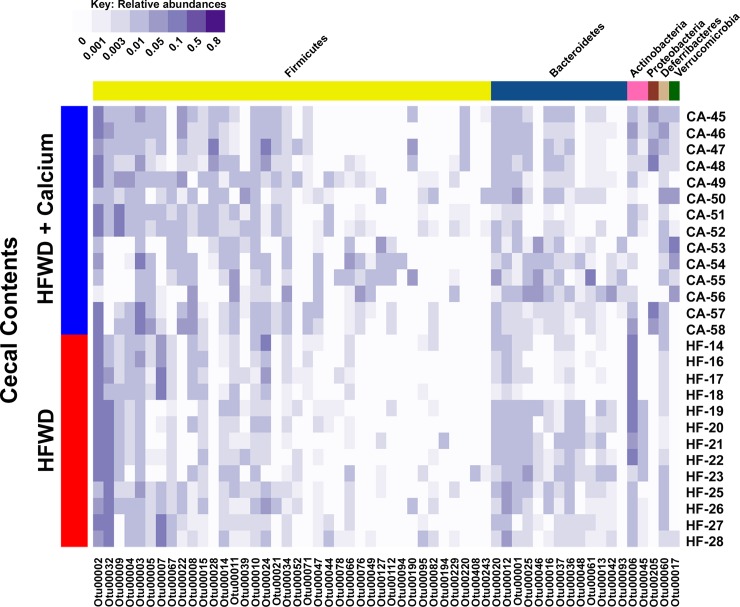
Microbial community composition within dietary groups. Cecal bacterial community compositions of individual samples. Each row represents the cecal bacterial community of one sample based on relative abundances of OTUs (97% sequence similarity) identified from V4 region sequences of 16S rRNA genes. Each column represents one OTU with darker shades of purple indicating higher relative abundances. OTUs were organized by taxonomic classification and only the OTUs that comprised at least 2% of one sample were included.

**Fig 6:**
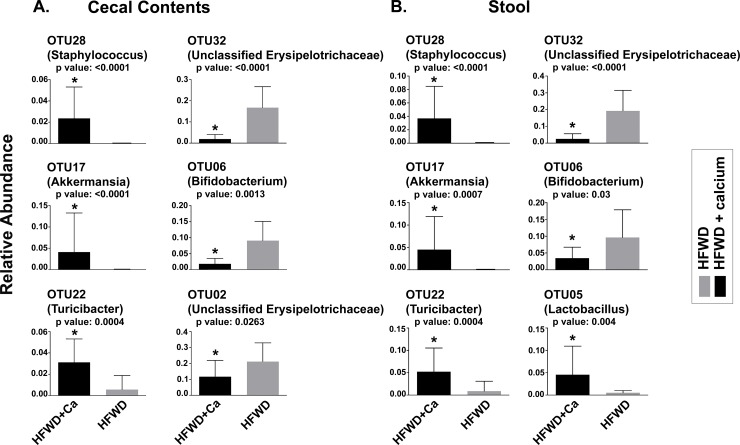
Relative abundance of selected taxa. Significant differences in relative abundance of major OTUs driving separation between HFWD-Ca and HFWD groups for cecal (A) and fecal (B) samples. Asterisks represent significance determined by Mann-Whitney U (p values above individual panels).

The overall microbial composition within individual samples from each dietary group is shown in Fig 5. Major OTUs driving the observed segregation between groups consisted of *Lactobacillus*, *Bifidobacterium*, and members of the *Erysipelotrichaceae*, and *Betaproteobacteria*, all elevated in the HFWD group; while the HFWD-Ca group had higher levels of *Streptococcus*, *Staphylococcus*, *Akkermansia*, *Turicibacter*, and *Ruminococcaceae* (Fig 6). Fecal and cecal alterations were similar.

Significant taxa driving differences were also evaluated by LEfSe [55]. A total of 51 differentiating features (ie. significantly different OTUs,) were identified using an LDA cut-off of 2 and a Wilcoxan p value of 0.05. These results were consistent with taxa driving separation in principal components analysis. The ten taxa with highest LDA scores for each dietary group are shown in Table 3. Taxa significantly elevated in the HFWD group consisted of members of *Erysipelotrichaceae*, *Betaproteobacteria*, and members of *Lachnospiraceae* (contains Lactobacillus spp). Taxa significantly elevated in the HFWD-Ca group included *Akkermansia*, *Turicibacter*, *and Staphylococcus*, as well as several members of *Lachnospiraceae* (different members than in HFWD group).

**Table 3:** Significant OTUs with ten highest LDA^a^ scores per diet group.

HFWD-Ca	LDA score	HFWD	LDA score
OTU17: *Akkermansia*	4.715	OTU32: unclassified *Erysipelotrichaceae*	5.016
OTU22: *Turicibacter*	4.528	OTU12: *Barnesiella*	4.154
OTU205: unclassified *Proteobacteria*	4.479	OTU45: *Olsenella*	3.790
OTU28: *Staphylococcus*	4.303	OTU36: unclassified *Bacteroidales*	3.570
OTU05: *Lactobacillus*	4.355	OTU95: unclassified *Lachnospiraceae*	3.464
OTU11: unclassified *Lachnospiraceae*	4.309	OTU74: unclassified *Betaproteobacteria*	3.405
OTU34: unclassified *Lachnospiraceae*	4.187	OTU13: unclassified *Porphyromonodaceae*	3.396
OTU112: unclassified *Lachnospiraceae*	3.879	OTU211: unclassified *Lachnospiraceae*	3.305
OTU16: unclassified *Porphyomonadaceae*	3.981	OTU153: unclassified *Lachnospiraceae*	3.088
OTU21:Clostridium XI	3.889	OTU261: unclassified *Lachnospiraceae*	3.082

^**a**^ Linear discriminant analysis

### Hepatic bile acid profiles

**Fig 7:**
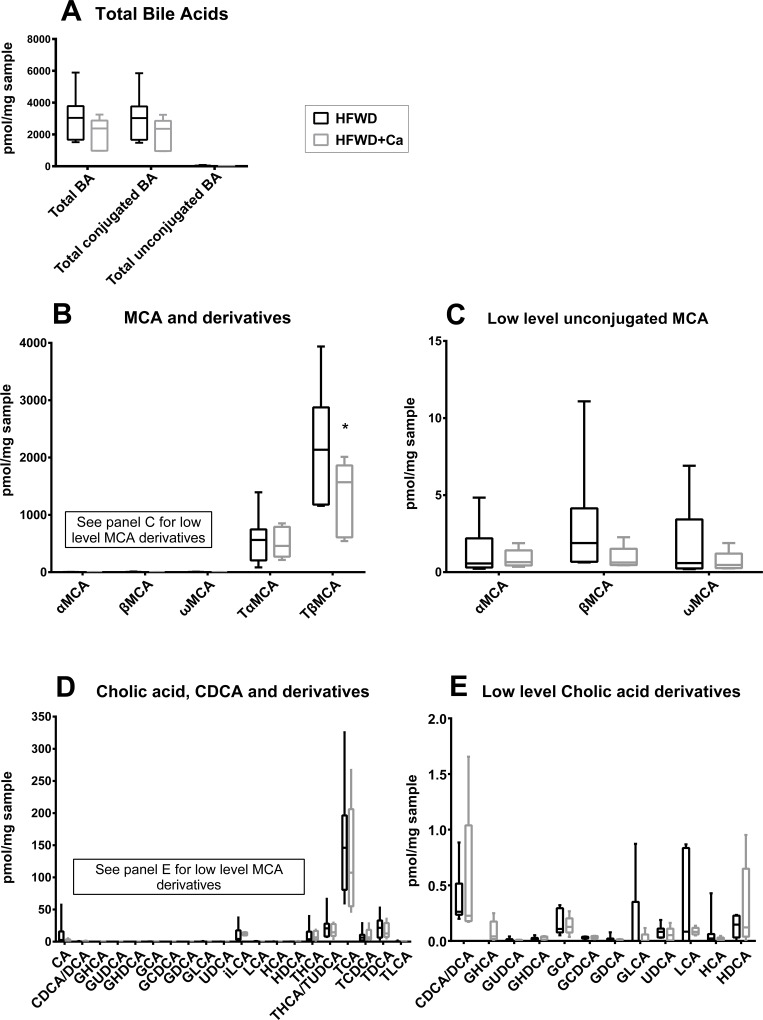
Hepatobiliary bile acid profiles. **A**. Total bile acids. Total bile acids and total conjugated bile acids were lower (but did not reach significance) in livers of mice from the HFWD-Ca group. **B**. Muricholic acid (MCA) and derivatives. TbMCA was significantly reduced in livers of HFWD-Ca mice (asterisk, p<0.00001, unpaired t test, Holm-Sidak correction for multiple comparisons). **C**. Low-prevalence MCA derivatives were also lower in the HFWD-Ca group, although this did not reach significance. **D**. Cholic acid (CA), chenodeoxycholic acid (CDCA) and derivatives. No differences between groups were seen (note y axis values indicating lower overall prevalence of CA/CDCA derivatives in mice in comparison to MCA). E. Low-prevalence CA derivatives. No differences between groups were seen. Data shown for all panels is from randomly selected mice without tumors (n = 7 for HFWD, n = 5 for HFWD-Ca).

Bile acid profiles were generated from samples of the left lateral and right median liver lobe (including gall bladder) of randomly selected 10 mice in the HFWD group and 9 mice in the HFWD-Ca group. Total bile acids and total conjugated bile acids were lower in the HFWD-Ca group, although this did not reach significance (Fig 7A). The major murine primary conjugated primary bile acid, tauro-β-muricholic acid (TbMCA), was significantly lower in the HFWD-Ca group (Fig 7B and 7C), both when mice with liver masses were excluded (p<0.00001) and when all samples were compared (p = 0.000015). There were no significant differences between groups in hepatic levels of cholic acid or its derivatives and conjugates (Fig 7D and 7E).

## Discussion

**Fig 8:**
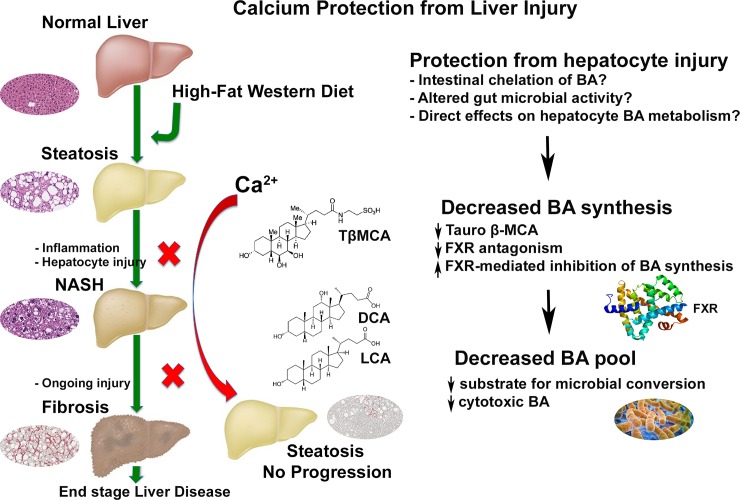
Proposed downstream site of calcium protection from liver injury. In the current study, calcium supplementation did not affect weight gain or hepatic steatosis, but did decrease the incidence of NASH-related histological changes, including fibrosis and regenerative hyperplasia associated with “end-stage” NASH. Thus, calcium supplementation could prevent the adverse downstream effects of obesity in situations where weight loss cannot be achieved or maintained. Although the mechanism is not known, potential factors could include gut chelation of bile acids or altered bile acid pools that may be related to gut microbial shifts. Calcium supplementation in this study was associated with decreased hepatic tauro-β- muricholic acid, which reduces FXR antagonism in the liver, permitting FXR-mediated inhibition of total bile acid synthesis. The decrease in hepatic total bile acid synthesis may decrease available substrate for downstream intestinal bacterial conversion of primary bile acids to cytotoxic secondary bile acids (LCA, DCA). Since a percentage of these bile acids are re-absorbed in the colon and transported to liver, the decrease in their synthesis could limit hepatic exposure to potentially cytotoxic BA molecular species.

This study evaluated the effects of dietary calcium on liver pathology in a murine model of high fat diet-induced NASH (ie. the histologic representation of NAFLD). Here we show that dietary calcium supplementation of male C57BL/6NCrl mice on a HFWD reduced liver injury over an 18-month period. Specifically, calcium supplementation i) decreased overall NAS (histological severity) and inflammation (Fig 1), ii) decreased liver fibrosis (Fig 2), iii) correlated with lower serum bilirubin and lower levels of the pro-inflammatory cytokine MCP-1 (Tables 1 and 2) and iv) decreased the number and size of “end-stage” hepatic regenerative hyperplastic nodules (Fig 3). Calcium has previously shown mixed results in protecting against weight gain in animals on high fat diets [28,30,31]. Other recent studies have demonstrated dietary-mediated changes in gastrointestinal microbes [58,59], and it has been suggested that changes in the microbiota influence weight gain. In our study, calcium-associated microbial changes were associated with improvement in liver structure/function, but did not affect weight gain. A limitation in the current study is that food consumption was not measured, however the lack of a significant weight difference suggests that the protective effects of calcium were not mediated by decreased weight gain. This suggests that calcium, rather than preventing obesity, may interfere with downstream progression from steatosis to NAFLD (Fig 8). This finding has potential clinical significance as it implies that calcium may protect against adverse progression even in those individuals unable to achieve or maintain weight loss, as is the case in many, if not most, human patients [60]. Additionally, the downstream events that mediate progression from steatosis to more serious manifestations of liver injury are not currently known, nor are the mechanisms by which dietary calcium may be protective. Two findings in this study that may provide mechanistic insight are altered gut microbial communities and decreases in the bile acid pool.

In this study, gut microbial communities in calcium-supplemented mice distinctly segregated from those of the non-supplemented mice (Figs 5 and 6). Significantly altered OTUs included several previously highlighted in other high fat diet studies. For example, HFWD-Ca mice had higher numbers of *Ruminococcaceae* and *Akkermansia*. *Ruminococcaceae* have been associated with a “healthy” gut microbiome, purportedly due to the ability to degrade complex plant polyglucans [61]. *Akkermansia* are associated with lipid metabolism capacity and inversely correlated with HFWD- induced obesity and metabolic dysregulation [62,63]. *Turicibacter*, also elevated in the HFWD-Ca group, has been associated with an anti-inflammatory phenotype [39]. In contrast, mice on the un-supplemented HFWD had higher levels of *Erysipelotrichaceae* and *Lactobacillus*, organisms associated with obesity [64,65]. A limitation of the current study is that it is not known whether these changes are merely correlative with calcium supplementation or whether the microbial alterations themselves affect liver health, perhaps through modulation of bile acid profiles.

With this in mind, we also found a calcium-related difference in hepatic bile acids (Fig 7). In high concentration, bile acids are cytotoxic [66]. Inhibition of biliary excretion and accumulation of bile acids in the liver (i.e. cholestasis) may contribute to progression of NASH [66]. In this study, the total hepatobiliary bile acid pool was lower in the HFWD-Ca mice than in HFWD controls. The majority of this reduction was in the amount of taurine-conjugated β-MCA (TbMCA), the major conjugated murine primary bile acid, as well as the level of the unconjugated parent β-MCA. TbMCA has FXR antagonist activity [67]. Bile acid synthesis in the liver is controlled through farnesoid X receptor (FXR) activation, which inhibits the rate-limiting enzyme in bile acid synthesis. Thus, in the HFWD-Ca mice, the lower levels of TbMCA (ie. reduced hepatic FXR antagonism) would favor FXR activity, thus suppressing bile acid synthesis. This is consistent with the overall decrease in the bile acid pool (observed here) and could in turn reduce downstream generation of potentially carcinogenic or cytotoxic secondary bile acids (Fig 8).

It is not known whether the calcium-associated alterations in bile acids and microbes seen here are independent or influenced by one another. Gut microbial populations are known to influence bile acid profiles and vice-versa [68]. Intestinal bacterial utilize 7α hydroxylase to convert primary bile acids to the secondary bile acids, including lithocholic acid (LCA) and deoxycholic acid (DCA). LCA is known to be cytotoxic to hepatocytes while DCA promotes cholestasis and gallstone formation [68]. Generation of LCA and DCA by 7α hydroxylase requires prior deconjugation by intestinal bacterial bile salt hydrolase (BSH). Although BSH activity was not measured in this study, taxa that were decreased in the HFWD-Ca group, including *Bifidobacterium*, some *Lactobacilli* spp, and some members of Bacteroidales are known to have BSH activity, [68]. Decreased relative abundance of these organisms may therefore decrease the conversion of the parent bile acids into cytotoxic secondary metabolites. Increased dietary calcium may also increase gut luminal precipitation of bile acids, decrease resorption, and increase bile acids in the soluble portion of feces [34,69–71]. Calcium may thus sequester potentially cytotoxic bile acids in the gut lumen and feces, acting similarly to pharmaceutical bile acid sequestrants currently used therapeutically against hyperlipidemia or type II diabetes[72]. Ultimately, alterations in gut microbial populations and altered bile acid pools may serve as a marker of dietary interventional efficacy, regardless of mechanistic role.

## Conclusions

In this study, dietary calcium supplementation mitigated downstream pathologic effects in a murine model of NASH, but did not affect steatosis or body weight. Calcium supplementation also correlated with significant differences in intestinal and fecal microbial communities and with decreased hepatic concentration of the primary conjugated murine bile acid (and hepatic FXR antagonist) taurine- β-MCA. These microbial and metabolic alterations may represent biomarkers of improved liver metabolic function or may play a mechanistic role in inhibiting the progression of NASH. The use of calcium supplementation as an interventional strategy for NAFLD progression is an attractive public health strategy, as dietary supplementation is easier to implement than sustained dietary change in human populations.

## Supporting Information

S1 Fig Mouse body weights (g) over time: Body weights over 18 months and endpoint liver weight. Male mice were maintained for 18 months on a high fat Western diet (20% fat) with and without calcium supplementation. Average body weights for mice in each diet group taken at 2 week intervals are shown. Differences between groups were not significant. Inset: Liver weights at necropsy. Inset: Mean liver weight from HFWD-Ca group was lower than that of HFWD group (p<0.05, unpaired t test).
(TIF)

S1 Table Diet used in this study. (DOCX)

S2 Table Raw data for in-life observations, histology, clinical pathology, cytokines. (XLSX)

S3 Table Bile acid data. (XLSX)

S4 Table Serum biochemical markers, all animals. (DOCX)

## Abbreviations

ALKP: Alkaline phosphatase
ALT: Alanine aminotransaminase
AST: Aspartate aminotransferase
ANOVA: Analysis of variance
HA: Hepatic adenoma
HCC: Hepatocellular carcinoma
H&E: Hematoxylin and eosin
HFWD: High-fat, Western style diet
NAFLD: Non-alcoholic fatty liver disease
NASH: Non-alcoholic steatohepatitis
RH: Regenerative hyperplasia
αMCA: α-Muricholate
βMCA: β-Muricholate
ωMCA: ω-Muricholate
TαMCA: α-tauro-Muricholate
TβMCA: β-tauro-Muricholate
CA: Cholate or Cholic acid
CDCA: DCA Chenodeoxycholic acid/Deoxycholic acid
GHCA: Glycohyocholic acid
GUDCA: Glycoursodeoxycholate
GHDCA: Glycol-hyodeoxycholate
GCA: Glycocholic acid
GCDCA: Glycochenodeoxycholate
GDCA: Glycodeoxycholate
GLCA: Glycolithocholate
UDCA: Urso deoxycholic acid
ILCA: Iso-Lithocholic acid
LCA: Lithocholic acid
HCA: Hyocholate
HDCA: Hyodeoxycholate
THCA: Tauro-hyocholate
THCA: TUDCA Tauro-hyocholate/Tauroursodeoxycholate
TCA: Taurocholic acid
TCDCA: Taurochenodeoxycholate
TDCA: Taurodeoxycholate
TLCA: Taurolithocholate
