# Correction: Calcium Reduces Liver Injury in Mice on a High-Fat Diet: Alterations in Microbial and Bile Acid Profiles

**DOI:** 10.1371/journal.pone.0170136

**Published:** 2017-01-09

**Authors:** Muhammad Nadeem Aslam, Christine M. Bassis, Li Zhang, Sameer Zaidi, James Varani, Ingrid L. Bergin

The first author’s name is incorrect in the citation. The correct citation is: Aslam MN, Bassis CM, Zhang L, Zaidi S, Varani J, Bergin IL (2016) Calcium Reduces Liver Injury in Mice on a High-Fat Diet: Alterations in Microbial and Bile Acid Profiles. PLoS ONE 11(11): e0166178.
