# Supplementary figures and images for: Calcium Reduces Liver Injury in Mice on a High-Fat Diet: Alterations in Microbial and Bile Acid Profiles
Source: PLoS One. 2016 Nov 16;11(11):e0166178. doi: 10.1371/journal.pone.0166178 (PMC5113033; doi:10.1371/journal.pone.0166178)

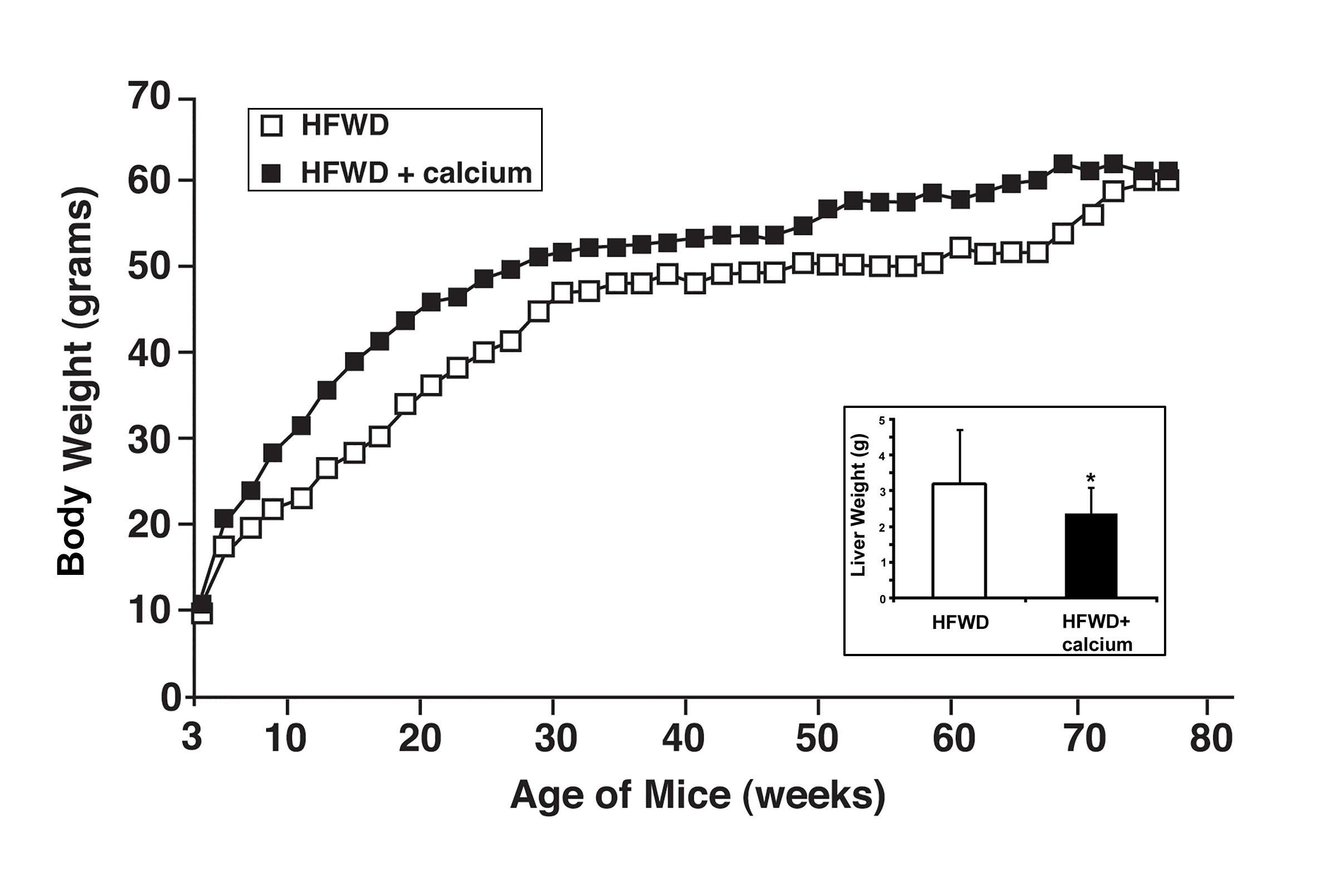

Supplement: S1 Fig — Male mice were maintained for 18 months on a high fat Western diet (20% fat) with and without calcium supplementation. Average body weights for mice in each diet group taken at 2 week intervals are shown. Differences between groups were not significant. Inset: Liver weights at necropsy. Inset: Mean liver weight from HFWD-Ca group was lower than that of HFWD group (p<0.05, unpaired t test). (TIF) [file pone.0166178.s001.tif]
