# Supplementary material for: Calcium Reduces Liver Injury in Mice on a High-Fat Diet: Alterations in Microbial and Bile Acid Profiles
Source: PLoS One. 2016 Nov 16;11(11):e0166178. doi: 10.1371/journal.pone.0166178 (PMC5113033; doi:10.1371/journal.pone.0166178)
Supplement: S1 Table — (DOCX) [file pone.0166178.s002.docx]

**S1 Table. Composition of the research diets used in the study.**

|  | | | | | |  |
| --- | --- | --- | --- | --- | --- | --- |
|  | | **HFWD** | | **HFWD + calcium** | |  |
|  | | **gm%** | **kcal%** | **gm%** | **kcal%** |  |
|  | |  |  |  |  |  |
| Protein | | 24.4 | 20.5 | 24.1 | 20.5 |  |
| Carbohydrate | | 49.7 | 41.8 | 49.1 | 41.8 |  |
| Fat | | 20 | 37.8 | 19.8 | 37.8 |  |
| Total | | 94.1 | 100 | 93.0 | 100 |  |
| kcal/gm | | 4.76 |  | 4.71 |  |  |
|  | |  |  |  |  |  |
| **Ingredients** | | **gm** | **kcal** | **gm** | **kcal** |  |
| Casein (80 Mesh) | | 240 | 960 | 240 | 960 |  |
| L-Cystine | | 3.6 | 14 | 3.6 | 14 |  |
| Corn starch | | 100 | 400 | 100 | 400 |  |
| Maltodextrin 10 | | 75 | 300 | 75 | 300 |  |
| Sucrose | | 310.418 | 1242 | 310.418 | 1242 |  |
| Cellulose (BW200) | | 20 | 0 | 20 | 0 |  |
| Corn oil | | 200 | 1800 | 200 | 1800 |  |
| Ethoxyquin | | 0.01 | 0 | 0.01 | 0 |  |
| Mineral mix S10001A^a^ | | 21 | 0 | 21 | 0 |  |
| Monosodium phosphate | | 7.98 | 0 | 7.98 | 0 |  |
| Monopotassium phosphate | | 7.91 | 0 | 7.91 | 0 |  |
| Calcium carbonate (40% calcium)^b^ | | 0.88 | 0 | 13 | 0 |  |
| Vitamin mix V13202 | | 12 | 48 | 12 | 48 |  |
| Choline bitartrate | | 1.2 | 0 | 1.2 | 0 |  |
| Folic acid | | 0.00023 | 0 | 0.00023 | 0 |  |
| Vitamin D3 (100,000 IU/g) | | 0.0012 | 0 | 0.0012 | 0 |  |
|  | |  |  |  |  |  |
| **Total** | | 1000.05 | 4764 | 1006.53 | 4764 |  |
|  |  |  |  |  |  |  |

^a^ Amount of essential minerals in mineral mix for these Western-style diets: magnesium oxide 0.5 gm/kg (60.3% Mg), chromium potassium sulfate 2 mg/kg (10.4% Cr), cupric carbonate 6 mg/kg (57.5% Cu), ferric citrate 45 mg/kg (21.2% Fe), manganous carbonate 59 mg/kg (47.8% Mn), sodium selenite 0.16 mg/kg (45.7%), zinc carbonate 29 mg/kg (52.1% Zn).

^b^ Amount of total calcium is 0.41 gm/kg in HFWD and 5.25 gm/kg in HFWD + calcium diet groups. Approximately 0.06 gm/kg of calcium is from Casein in both diets.
