# Supplementary material for: Calcium Reduces Liver Injury in Mice on a High-Fat Diet: Alterations in Microbial and Bile Acid Profiles
Source: PLoS One. 2016 Nov 16;11(11):e0166178. doi: 10.1371/journal.pone.0166178 (PMC5113033; doi:10.1371/journal.pone.0166178)
Supplement: S4 Table — (DOCX) [file pone.0166178.s005.docx]

S4 Table. Serum biomarkers in diet groups, including animals with liver masses^a,b^

|  | HFWD (n=15) | HFWD/Ca (n=16) |
| --- | --- | --- |
| AST | 167 ± 125 | 153 ± 74 |
| ALT | 137 ± 185 | 87 ± 40 |
| AlkP | 63 ± 28 | 46 ± 49 |
| Total bilirubin | 0.13 ± 0.06 | 0.07 ± 0.06* |
| Albumin | 3.06 ± 0.56 | 3.05 ± 0.50 |
| Total protein | 5.86 ± 0.91 | 6.15 ± 0.83 |
| Creatinine | 0.19 ± 0.04 | 0.18 ± 0.03 |
| Glucose | 273 ±80 | 247 ± 60 |
| Calcium | 9.56 ± 1.17 | 9.85 ± 0.71 |

^a^ A total of 20 animals were present in each group. Serum values were not available from 5 HFWD and 4 Ca HFWD animals owing to post-mortem coagulation or insufficient sample.

^b^ Values are mean +/- S.D. *p=0.0116, unpaired t test, two-tailed without multiple comparisons correction.

ALKP alkaline phosphatase, ALT alanine aminotransaminase, AST Aspartate Aminotransferase.
